# Comparative Morpho-Physiological, Biochemical, and Gene Expressional Analyses Uncover Mechanisms of Waterlogging Tolerance in Two Soybean Introgression Lines

**DOI:** 10.3390/plants13071011

**Published:** 2024-04-02

**Authors:** Ripa Akter Sharmin, Benjamin Karikari, Mashiur Rahman Bhuiyan, Keke Kong, Zheping Yu, Chunting Zhang, Tuanjie Zhao

**Affiliations:** 1 Key Laboratory of Biology and Genetics Improvement of Soybean, Ministry of Agriculture, Zhongshan Biological Breeding Laboratory (ZSBBL), National Innovation Platform for Soybean Breeding and Industry-Education Integration, State Key Laboratory of Crop Genetics & Germplasm Enhancement and Utilization, College of Agriculture, Nanjing Agricultural University, Nanjing 210095, China; 2 Department of Botany, Jagannath University, Dhaka 1100, Bangladesh; 3 Department of Crop Science, Faculty of Agriculture, Food and Consumer Sciences, University for Development Studies, Tamale P.O. Box TL 1882, Ghana; 4 Département de Phytologie, Université Laval, Québec, QC G1V 0A6, Canada; 5 Institute of Horticulture, Zhejiang Academy of Agricultural Sciences, Hangzhou 310021, China

**Keywords:** soybean, waterlogging, morphological difference, antioxidant activity, gene expression

## Abstract

Waterlogging is one of the key abiotic factors that severely impedes the growth and productivity of soybeans on a global scale. To develop soybean cultivars that are tolerant to waterlogging, it is a prerequisite to unravel the mechanisms governing soybean responses to waterlogging. Hence, we explored the morphological, physiological, biochemical, and transcriptional changes in two contrasting soybean introgression lines, A192 (waterlogging tolerant, WT) and A186 (waterlogging sensitive, WS), under waterlogging. In comparison to the WT line, waterlogging drastically decreased the root length (RL), shoot length (ShL), root fresh weight (RFW), shoot fresh weight (ShFW), root dry weight (RDW), and shoot dry weight (ShDW) of the WS line. Similarly, waterlogging inhibited soybean plant growth by suppressing the plant’s photosynthetic capacity, enhancing oxidative damage from reactive oxygen species, and decreasing the chlorophyll content in the WS line but not in the WT line. To counteract the oxidative damage and lipid peroxidation, the WT line exhibited increased activity of antioxidant enzymes such as peroxidase (POD), superoxide dismutase (SOD), and catalase (CAT), as well as higher levels of proline content than the WS line. In addition, the expression of antioxidant enzyme genes (*POD1*, *POD2*, *FeSOD*, *Cu*/*ZnSOD*, *CAT1*, and *CAT2*) and ethylene-related genes (such as *ACO1*, *ACO2*, *ACS1*, and *ACS2*) were found to be up-regulated in WT line under waterlogging stress conditions. In contrast, these genes showed a down-regulation in their expression levels in the stressed WS line. The integration of morpho-physiological, biochemical, and gene expression analyses provide a comprehensive understanding of the responses of WT and WS lines to waterlogging conditions. These findings would be beneficial for the future development of soybean cultivars that can withstand waterlogging.

## 1. Introduction

Soybean, as the world’s leading leguminous oilseed crop, plays a crucial role in providing vegetable oil and protein to both humans and animals. With a global grain production of approximately 359.79 million tons (United States Department of Agriculture, 2021–2022), soybeans are of great economic significance. However, waterlogging poses a significant challenge for soybean cultivation at various stages of development, including the vegetative and reproductive stages [1,2]. Waterlogging is a widespread problem that has a detrimental impact on soybean development and yield in numerous regions across the globe. This is often caused by excessive rainfall or irrigation without proper drainage, leading to transient or prolonged water accumulation in the soil [3]. Waterlogging is considered the second most impactful abiotic stress factor in soybeans after drought. It can lead to yield losses of up to 80% [4]. For instance, it decreased soybean yields by 17% and 50% during the vegetative and reproductive stage, respectively [5]. It inhibited the uptake of nutrient, particularly nitrogen and magnesium, leading to negative impacts on soybean growth, metabolism, photosynthesis, and yield [6].

In addition to the effects mentioned above, waterlogging damages plant roots, which alters leaf physiology and impacts the concentration of chlorophyll (Chl), transpiration rate (Tr), net photosynthetic rate (Pn), stomatal conductance (gs), and net CO_2_ assimilation rate (Ci) [7]. It also restricts the growth and biomass accumulation of plants due to decreased CO_2_ accumulation and limited uptake of water and nutrients [8]. The overproduction of reactive oxygen species (ROS) and redox imbalance is another detrimental effect of waterlogging on agricultural production losses [9]. The excess ROS react with unsaturated fatty acids in membranes, leading to the production of lipid hydroperoxides and malondialdehyde (MDA), which cause membrane damage and disrupt cell integrity [10].

To counteract the negative impacts of waterlogging, plants employ sophisticated signaling pathways to regulate the activation of stress-responsive genes, resulting in a variety of adaptations at the morphological, physiological, and biochemical levels [11,12,13]. In response to waterlogging stress, plants undergo modifications in their root structure such as the emergence of adventitious roots (ARs). These changes serve to alleviate the oxidative stress and improve oxygen diffusion to the roots under hypoxic conditions [13]. Plants that are coping with waterlogging stress usually grow adventitious roots, which can aid in better nutrient and water intake as well as gas transmission [14]. Moreover, plants have evolved an effective antioxidative defense strategy to counteract the accumulation of excess ROS and protect themselves from oxidative damage under waterlogging conditions. This defense mechanism relies on a range of enzymes, like POD, CAT, SOD, glutathione peroxidase (GPX), glutathione reductase (GR), and ascorbate peroxidase (APX), as well as non-enzymatic antioxidants including glutathione (GSH), ascorbate (AsA), and phenols [10,11]. Their activities help neutralize excessive ROS levels generated due to hypoxia-induced metabolic changes, preventing oxidative damage to essential cellular components [15]. Previous research has shown that genes related to ethylene biosynthesis and antioxidant enzyme genes are essential for enhancing waterlogging tolerance. For instance, the expression of ethylene biosynthesis genes (S-adenosyl-L-methionine (SAM) and 1-aminocyclopropane-1-carboxylic acid oxidase (ACCO)) showed an up-regulation in banana plants subjected to waterlogging stress [16]. Additionally, it has been reported that genes involved in ROS scavenging are up-regulated in the leaves of rape seedlings when subjected to waterlogging [17].

To date, the adaptation mechanisms underlying waterlogging tolerance in soybean remain largely unexplored. An in-depth understanding of waterlogging tolerance mechanisms is essential to improving soybean’s response to waterlogging by leveraging advances made in other crops such as rice, maize, wheat, and tomato [18,19,20,21]. Thus, the present study aimed to assess the morpho-physiological, biochemical, and gene expression responses to waterlogging in two soybean lines with contrasting responses: A192 (waterlogging tolerant, WT) and A186 (waterlogging sensitive, WS). The findings from this study would be valuable in breeding climate-smart soybean cultivars, particularly those with waterlogging tolerance.

## 2. Results

### 2.1. Morphological Differences between WT and WS Soybean Lines with and without Waterlogging

**Figure 1:**
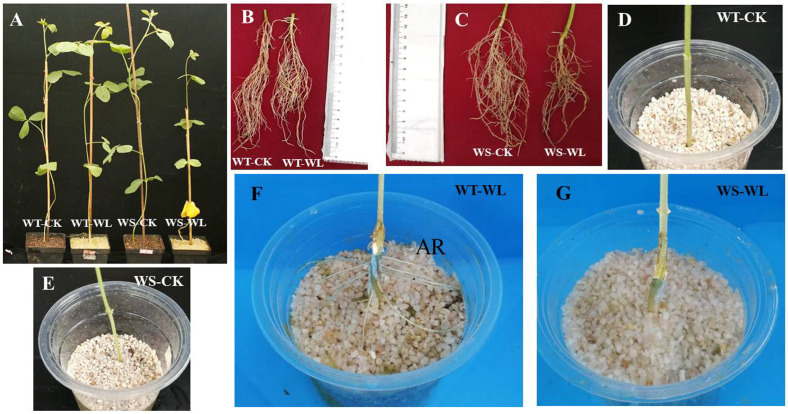
Phenotype of A192 (waterlogging tolerant, WT) and A186 (waterlogging sensitive, WS) soybean lines grown under control and waterlogging conditions for 21 days. (**A**) Growth performance of WT and WS soybean plants under control and waterlogging conditions. (**B**) WT roots under control and waterlogging conditions. (**C**) WS roots under control and waterlogging. (**D**) WT control. (**E**) WS control. (**F**) Adventitious root (AR) formation in the WT plants. (**G**) No AR formation in WS plants. WT- and WS-CK represent the control condition where plants received regular amounts of water, while WT- and WS-WL represent waterlogging conditions where a 21-day waterlogging was imposed by maintaining the water level at more than 3 cm above the soil surface.

To investigate the waterlogging tolerance of the WT and WS soybean lines, we examined various phenotypic traits (viz., RL, ShL, RFW, ShFW, RDW, and ShDW) after subjecting the soybean plants to 21 days of waterlogging (Table 1). The results showed significant differences between the two soybean lines under waterlogging conditions (*p* < 0.05). Specifically, the WS line exhibited a more pronounced adverse effect on plant growth under waterlogging compared to the WT line, as evidenced by significant differences in RL, ShL, RFW, ShFW, RDW, and ShDW (Figure 1A–C; Table 1). Moreover, we observed that the WT line displayed well-developed adventitious roots under waterlogging stress, whereas the WS line completely lacked adventitious roots (Figure 1D–G). The contrasting responses of the WT and WS lines to waterlogging provide valuable insights into their unique characteristics and make them ideal candidates for studying and comprehending the underlying mechanisms of waterlogging tolerance.

### 2.2. Physiological Differences between WT and WS Lines in Response to Waterlogged Conditions

**Figure 2:**
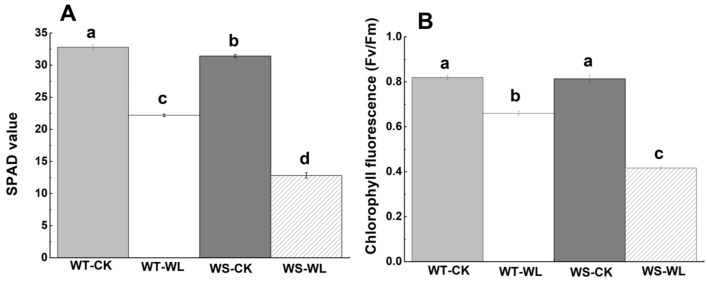
Changes in chlorophyll contents and chlorophyll fluorescence in A192 (waterlogging tolerant, WT) and A186 (waterlogging sensitive, WS) soybean lines under control and waterlogging conditions. (**A**) Chlorophyll contents (SPAD value). (**B**) Chlorophyll fluorescence. The bars in the graph represent the mean ± standard error of three replicates. Different letters in different columns indicate statistical significance, as determined by Duncan’s Multiple Range Test with *p* < 0.05. WT- and WS-CK represent the control condition where the plants received regular amounts of water, while WT- and WS-WL represent waterlogging conditions.

**Figure 3:**
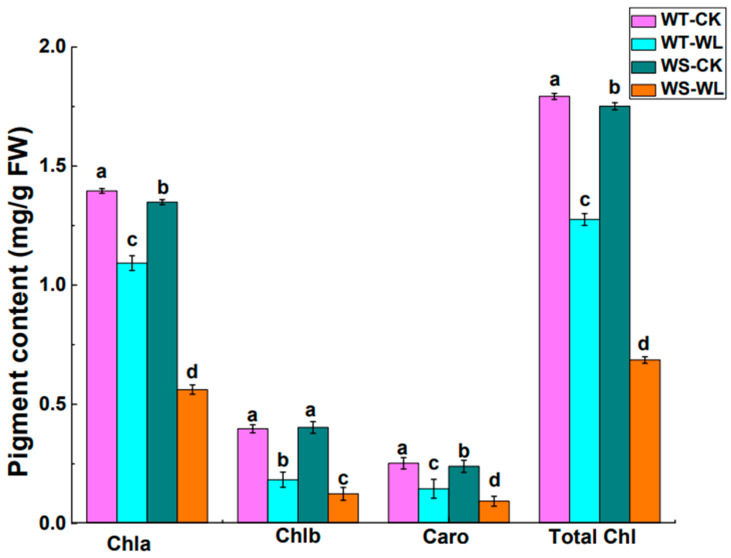
Comparison of leaf photosynthetic pigment contents in A192 (waterlogging tolerant, WT) and A186 (waterlogging sensitive, WS) soybean lines under control and waterlogging conditions. Chl a, chlorophyll a (mg/g FW); Chl b, chlorophyll b (mg/g FW); Caro, carotenoids (mg/g FW); Total Chl, total chlorophyll (mg/g FW). FW represents fresh weight. The bars in the graph represent the mean ± standard error of three replicates. Different letters in different columns indicate statistical significance, as determined by Duncan’s Multiple Range Test with *p* < 0.05. WT- and WS-CK represent the control condition where the plants received regular amounts of water, while WT- and WS-WL represent waterlogging conditions.

**Figure 4:**
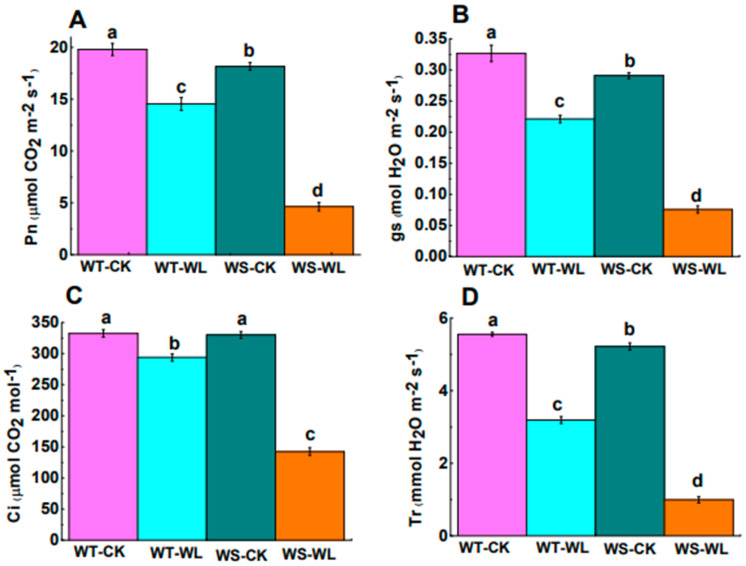
Gas exchange parameters of A192 (waterlogging tolerant, WT) and A186 (waterlogging sensitive, WS) soybean lines under control and waterlogging conditions. (**A**) Net photosynthesis rate (Pn). (**B**) Stomatal conductance (gs). (**C**) Net CO_2_ assimilation rate (Ci). (**D**) Transpiration rate (tr). The bars in the graph represent the mean ± standard error of three replicates. Different letters in different columns indicate statistical significance, as determined by Duncan’s Multiple Range Test with *p* < 0.05. WT- and WS-CK represent the control condition where the plants received regular amounts of water, while WT- and WS-WL represent waterlogging conditions.

Waterlogging caused a significant decline in all physiological activities in both the WT and WS lines in comparison to control plants (Figure 2, Figure 3 and Figure 4). The Chl content in both the WT and WS lines was assessed using a SPAD meter to monitor changes in physiological parameters under waterlogged conditions. The analysis of the Chl content revealed a notable reduction in accumulation in the WS line compared to the WT line (Figure 2A). To assess how the different light systems changed in response to waterlogging, we measured the maximum photochemical efficiency (Fv/Fm). In Figure 2B, it can be observed that the Fv/Fm values were higher in the WT and WS soybean plants that were not exposed to waterlogging. The chlorophyll fluorescence in the WT soybean line was higher than that of the WS line (Figure 2B). Furthermore, the pigment composition of the leaves, including Chl a, Chl b, and carotenoids, was analyzed in both the WT and WS lines to evaluate alternations in pigment composition under waterlogging conditions. The WS line showed a reduction in the levels of Chl a, Chl b, carotenoids, and total chlorophyll of photosynthetic pigments compared to the WT line (Figure 3).

We also examined gas exchange indicators such as Pn, gs, Ci, and Tr under both normal and waterlogged conditions to assess the photosynthetic capacity of the WT and WS soybean lines. In comparison to the control plants, both the WT and WS soybean plants displayed a reduction in all of these gaseous characteristics under waterlogged conditions. The Pn in the WT line was recorded as 14.54 μmol m^−2^ s^−1^, which was higher than that of the WS line, which measured 4.63 μmol m^−2^ s^−1^ (Figure 4A). Likewise, gs, Ci, and Tr were also notably higher in the WT line compared to the WS line (Figure 4B–D). These results showed that under waterlogging treatment, the photosynthetic capability of the WT line remained higher than that of the WS line. These could be the basis for the better performance of the WT line compared to the WS line under waterlogging conditions.

### 2.3. Cellular Membrane Integrity under Waterlogging Conditions

**Figure 5:**
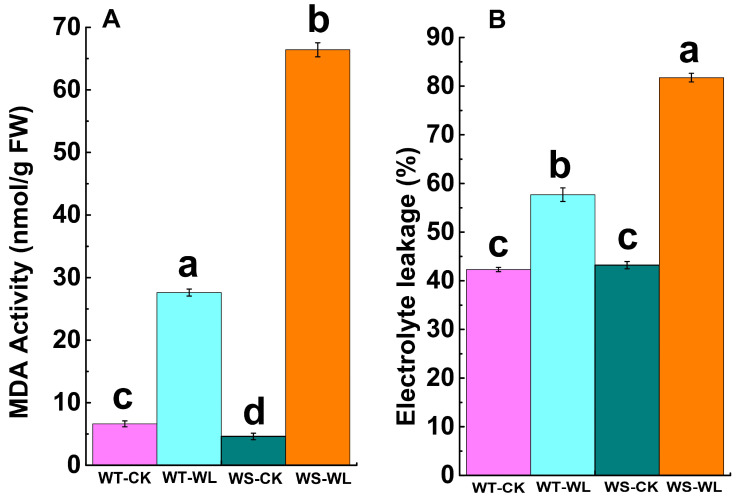
Comparison of malondialdehyde (MDA) content and electrolyte leakage (EL) in the leaves of A192 (waterlogging tolerant, WT) and A186 (waterlogging sensitive, WS) lines under control and waterlogging conditions. (**A**) Malondialdehyde (MDA) content. (**B**) Electrolyte leakage (EL). The bars in the graph represent the mean ± standard error of three replicates. Different letters in different columns indicate statistical significance, as determined by Duncan’s Multiple Range Test with *p* < 0.05. WT- and WS-CK represent the control condition where plants received regular amounts of water, while WT- and WS-WL represent waterlogging conditions.

**Figure 6:**
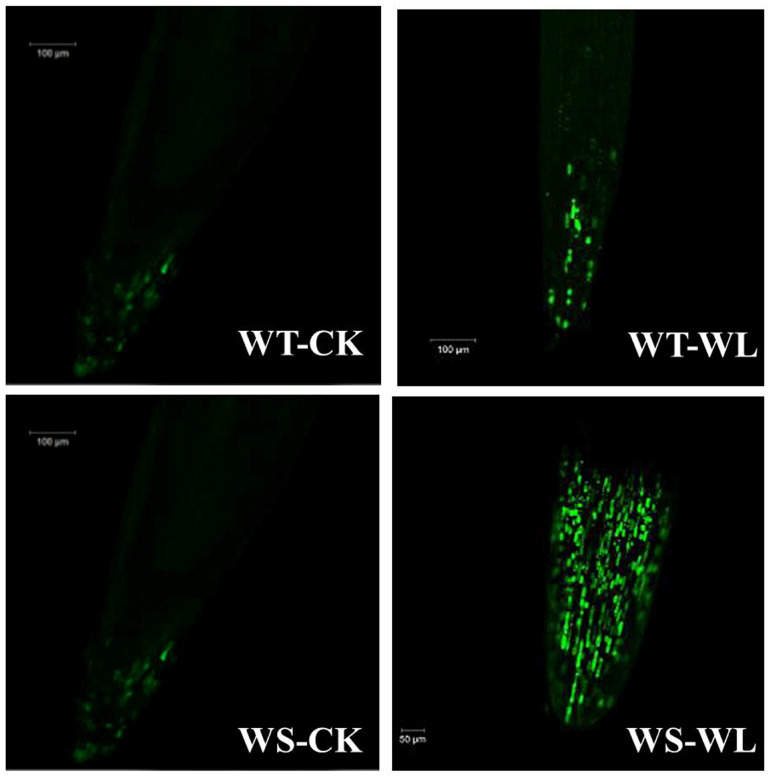
Reactive oxygen species (ROS) production in the meristematic zone of roots in A192 and A186 soybean lines under control and waterlogging conditions. Visualization of ROS (H_2_O_2_) production under green light (480–550 nm) fluorescence with a confocal microscope. WT- and WS-CK represent the control condition where plants received regular amounts of water, while WT- and WS-WL represent waterlogging conditions.

To investigate the damage to cell membranes, we quantified the MDA content and leaf electrolyte leakage (EL) in the leaves of the WT and WS soybean lines under normal and waterlogging conditions. The WS plants exhibited significantly higher levels of MDA (14.43-fold increase) and EL (1.86-fold increase) under waterlogging compared to control conditions (Figure 5A,B). In contrast, the WT plants showed smaller changes in MDA and EL levels after the same duration of waterlogging stress (Figure 5A,B). These findings indicate that the WT plants enhanced their tolerance to waterlogging due to their ability to maintain cellular membrane stability and integrity by reducing the production of MDA and EL. For further insights, we visualized the ROS in the roots of the WT and WS soybean lines using 2′,7′-dichlorofluorescin diacetate. Confocal microscopy images revealed the presence of H_2_O_2_ within the meristematic zone of both WT and WS roots. Notably, waterlogged WS roots exhibited higher amounts of H_2_O_2_ than waterlogged WT roots, even though control WS roots also showed H_2_O_2_ (Figure 6).

### 2.4. Changes in Antioxidant Defense System under Waterlogging Condition

**Figure 7:**
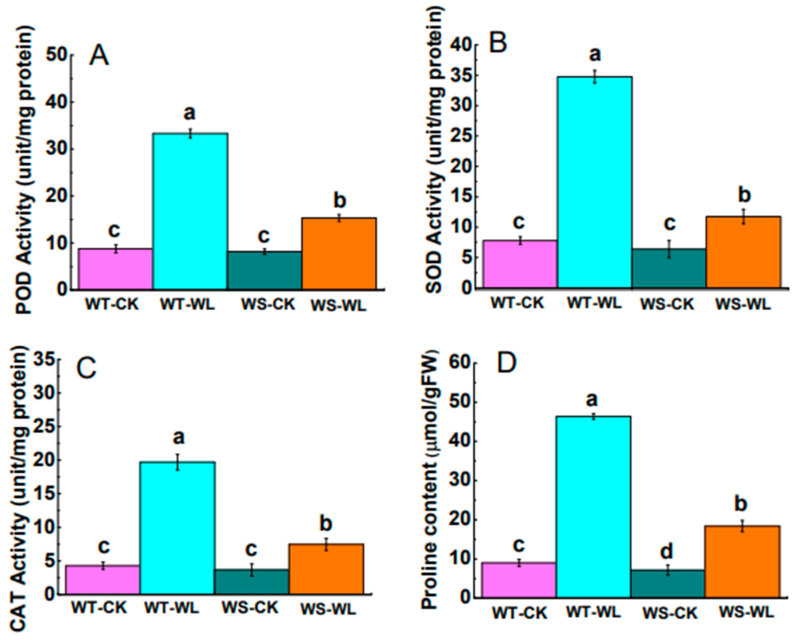
ROS-scavenging enzyme activities and proline content in the leaves of A192 (waterlogging tolerant, WT) and A186 (waterlogging sensitive, WS) soybean lines under control and waterlogging conditions. (**A**) POD activity; (**B**) SOD activity; (**C**) CAT activity; (**D**) proline content. Different letters in different columns indicate statistically significant differences, according to Duncan’s Multiple Range Test with *p* < 0.05. The bars represent the mean ± standard error of three replicates. WT- and WS-CK represent the control condition where plants received regular amounts of water, while WT- and WS-WL represent waterlogging conditions.

To understand the adaptive mechanism of waterlogging tolerance, we determined the POD, SOD, and CAT enzyme activities, along with proline content in both the WT and WS lines. As shown in Figure 7A–C, significantly higher POD, SOD, and CAT activities were detected in the WT line compared to the WS line. In Figure 7D, it can be observed that the WT soybean plants exposed to waterlogging stress had a significant increase in the accumulation of proline relative to the WS plants under waterlogging. These results suggest that the tolerant WT line reduced oxidative damage by stimulating the activities of the antioxidant enzymes.

### 2.5. Gene Expression Analysis of Waterlogging-Responsive Genes

**Figure 8:**
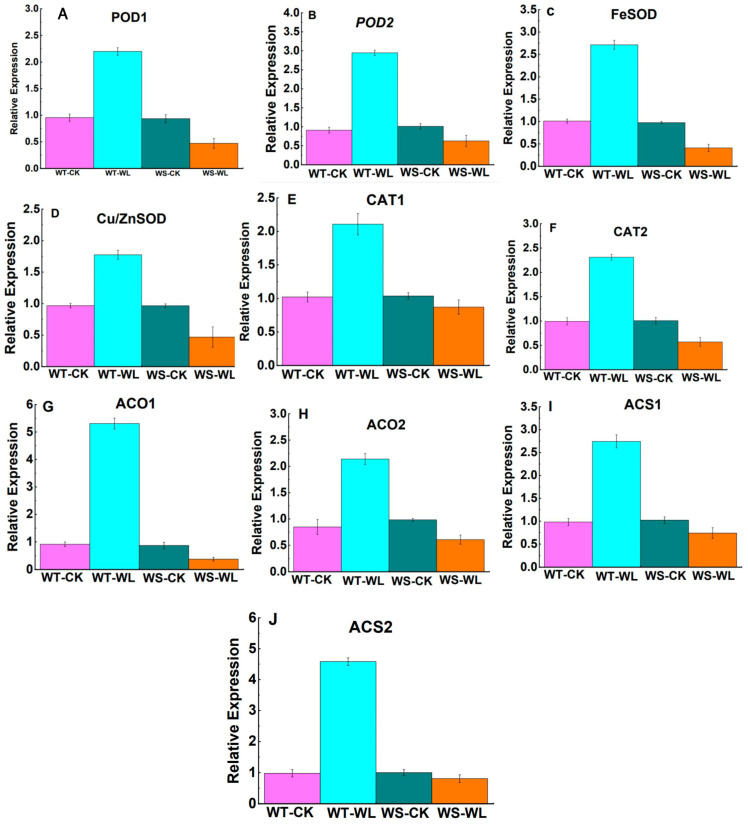
Relative expression levels of ten possible candidate genes associated with waterlogging tolerance in the roots of A192 (waterlogging tolerant, WT) and A186 (waterlogging sensitive, WS) soybean lines. (**A**) *POD1*, (**B**) *POD2*, (**C**) *FeSOD*, (**D**) *Cu*/*ZnSOD*, (**E**) *CAT1*, (**F**) *CAT2*, (**G**) *ACO1*, (**H**) *ACO2*, (**I**) *ACS1*, and (**J**) *ACS2*.

The expression of waterlogging-responsive genes was measured in both the WT and WS lines under waterlogging stress and normal conditions (Figure 8A–J). The genes *POD1* (*Glyma.02G008900*), *POD2* (*Glyma.17G053000*), *FeSOD* (*Glyma.02G087700*), *Cu/ZnSOD* (*Glyma.11G192700*), *CAT1* (*Glyma.04G017500*), and *CAT2* (*Glyma.17G261700*) were up-regulated by 2.30-, 3.23-, 2.68-, 1.83-, 2.06-, and 2.32-fold, respectively, in the waterlogged WT line compared to its counterpart under normal conditions (Figure 8A–F). In contrast, these genes exhibited down-regulation in the waterlogged WS line compared to the control WS line (Figure 8A–F). Additionally, the transcript abundance of four ethylene-related genes was quantified in both the WT and WS lines under both conditions. The genes *ACO1* (*Glyma.04G245900*), *ACO2* (*Glyma.09G255000*), *ACS1* (*Glyma.05G223000*), and *ACS2* (*Glyma.08G030100*) in the WT line showed significantly higher expression under waterlogging compared to the control condition. Specifically, *ACO1* was increased by 5.8-fold, *ACO2* by 2.52-fold, *ACS1* by 2.79-fold, and *ACS2* by 4.6-fold (Figure 8G–J). However, these genes were down-regulated in the waterlogged WS line. Notably, among these genes, *ACO1* and *ACS2* had higher expression levels in the WT line compared to the WS line under waterlogging.

## 3. Discussion

### 3.1. WT Line Grows Better under Waterlogging Than WS Line

Waterlogging is a highly destructive abiotic stress factor that has detrimental effects on soybean plants through root damage, disrupting photosynthesis, impairing antioxidant defense mechanisms, and potentially leading to plant death [8,22]. To study the waterlogging stress responses of two different soybean lines, A192 (WT) and A186 (WS), we examined various morphological, physiological, and biochemical indicators after subjecting the plants to 21 days of waterlogging treatment. Our findings showed a substantial reduction in RL, ShL, RFW, ShFW, RDW, and ShDW in both the WT and WS lines compared to the control plants; however, the degree of reduction was higher in the WS line than in the WT line (Table 1). Hence, the WS waterlogging-treated plants are affected by waterlogging to a greater degree, whereas waterlogged WT plants showed minimal phenotypic changes by retaining fully extended green leaves and an undamaged plant architecture. These findings are in agreement with earlier research showing that waterlogging results in severe root and shoot growth inhibition [3,18]. Therefore, these morphological traits are crucial for screening flood-tolerant soybean varieties under normal conditions.

The roots are an underground organ of a plant that play a vital function in absorbing water and minerals for plant development and yield productivity, which are influenced by various abiotic and biotic stresses in soils [23]. Taproots with a greater density of lateral roots were observed in the WT line, which enabled its adaptation to waterlogging compared with the WS line (Figure 1B). Waterlogging conditions trigger AR formation through the activity of plant hormones, ethylene, auxin, and cytokinin [24,25,26,27]. The emergence of ARs at the base of the shoot is a notable plant response to waterlogging stress [14]. The WT line developed more adventitious roots under the waterlogging treatment, whereas adventitious roots were absent in the WS soybean line. Therefore, adventitious root formation under waterlogging might be an adaptive mechanism in the tolerant line.

### 3.2. WT Line Had Better Photosynthetic Performance than WS Line under Waterlogging Stress

Elucidating the physiological changes has become extremely important for the evaluation of soybean waterlogging tolerance. Waterlogging causes a significant reduction in the photosynthesis process by altering the concentration of different pigments and metabolites, the ultrastructure of organelles, and the regulation of stomata [28,29,30]. The Chl content can be reduced due to waterlogging, which can produce visible symptoms like browning or yellowing of the leaves. This reduction in chlorophyll levels hinders photosynthesis, which has an adverse effect on plant growth and development [1]. For example, a susceptible genotype of sesame (Ezhi-2) had a decreased in Chl content while the tolerant genotype (ZZM2541) exhibited minimal alterations in total Chl levels [31]. In the current study, the WT line showed a gradual decline in Chl content in response to waterlogging compared to the WS line (Figure 2A). Moreover, photosynthetic pigments play a vital role in plant photosynthesis, and any changes in their levels directly affect the photosynthetic activity [32]. The WT line exhibited a significantly higher content of photosynthetic pigments, such as Chl a, Chl b, carotenoids, and total Chl content, in comparison to the WS line. The reductions in these photosynthetic pigments due to waterlogging led to a decrease in photosynthetic capacity and this aligns with the results from previous research on soybean [1]. Furthermore, Chl fluorescence parameters are adversely affected by stress, indicating that the photosynthetic processes are functioning less efficiently [33]. The Chl fluorescence in the WT soybean line was higher than that in the WS line in our findings (Figure 2B).

A reduced gs was the primary factor limiting photosynthesis, as seen by the fall in Pn, gs, and Ci in response to waterlogging [34]. Photosynthesis in the WS plants decreased significantly compared to WT plants. This finding demonstrated that the WT line sustained CO_2_ fixation and assimilation. For these reasons, the Chl content in the WT line was only slightly affected under waterlogging, which is consistent with recent findings in rice [18]. Collectively, these findings suggested that an increased photosynthetic capacity could be an adaptive indicator in terms of waterlogging tolerance.

### 3.3. WT Line Maintained Cellular Membrane Integrity and Exhibited a Robust Antioxidant System under Waterlogging Stress

In optimal conditions, plants maintain a delicate equilibrium between the generation and elimination of ROS. However, when plants are subjected to stress such as waterlogging, this balance is disrupted [35,36]. As a consequence of waterlogging conditions, the cell membrane is disturbed, leading to an elevation in ROS production. One specific ROS, H_2_O_2_, damages membrane lipids and leads to increased levels of lipid peroxidation [37,38]. MDA is a useful indicator for measuring lipid peroxidation in stressed plants. Previous research showed a notable increase in MDA accumulation in plants subjected to waterlogging stress [10]. The waterlogged WS line had significantly elevated levels of MDA and EL, indicating more extensive membrane damage compared to the WT line in our study (Figure 5A,B). Waterlogged WS roots showed higher amounts of H_2_O_2_ compared to waterlogged WT roots, although control WS roots also displayed H_2_O_2_ (Figure 6). The increased ROS concentration in WS roots indicates that they are susceptible to soil flooding.

To counter oxidative stress and maintain cellular activity, plants regulate the expression of scavenger enzymes such as SOD, POD, CAT, and APX in the cell membrane system to counteract oxidative damage. This regulatory mechanism is considered a significant pathway for enhancing tolerance to adverse conditions [39,40,41]. The interaction between enzymatic and non-enzymatic antioxidants helps to maintain equilibrium and cellular ROS stability to protect plants from oxidative damage [11,12]. Among these enzymes, SOD plays a crucial role in scavenging ROS and serves as the primary defense mechanism in the antioxidant system; it resolves H_2_O_2_, which is then rapidly detoxified by CAT, leading to the conversion of H_2_O_2_ into water and oxygen [41]. Subsequently, other enzymes such as POD and APX also contribute to detoxifying the H_2_O_2_ in plants. Bansal et al. [42] found that waterlogging-resistant plants showed significantly higher antioxidant enzyme activity compared to waterlogging-sensitive plants. Our results indicated that the WT line had higher levels of POD, SOD, and CAT activities compared to the WS line under waterlogging (Figure 7A–C). Moreover, genes encoding antioxidant enzymes play a crucial role in enhancing waterlogging tolerance. For instance, a gene expression analysis in mung bean revealed that the roots of waterlogged V. luteola and T 44 plants exhibited increased expression levels of *Cu*/*ZnSOD* and *APX*. In contrast, minimal expression of these genes was observed in the control or treated plants of Pusa Baisakhi [43]. Antioxidant-related genes, such as *POD1*, *POD2*, *FeSOD*, *Cu*/*ZnSOD*, *CAT1*, and *CAT2*, were up-regulated in the waterlogged WT line in our study.

Proline, an essential amino acid, is crucial for plants to respond to stress [44,45]. In this study, it was found that the WT soybean plants accumulated higher levels of proline compared to the WS plants. This is consistent with previous studies on other plant species such as barley [46], tomato [47], and cucumber [48]. The accumulation of proline in plants under waterlogging stress may help them maintain their water status and hydraulic conductivity by acting as an osmolyte. In addition, ethylene acts as a signaling molecule during flooding events. This was corroborated by a transcriptome analysis that revealed the up-regulation of genes associated with ethylene biosynthesis in submerged roots [16,49]. Hu et al. [50] reported forty differentially expressed genes involved in ethylene signaling in mulberry plants under waterlogging. This finding emphasizes the important role of phytohormones in modulating the signaling networks that respond to waterlogging in plants. Our results indicated that the expression of genes involved in ethylene biosynthesis (*ACO1*, *ACO2*, *ACS1*, and *ACS2*) were up-regulated in the WT line under waterlogging relative to WS line (Figure 8), indicating that the WT line has better tolerance to waterlogging due to the activation of stress-responsive genes.

## 4. Conclusions

**Figure 9:**
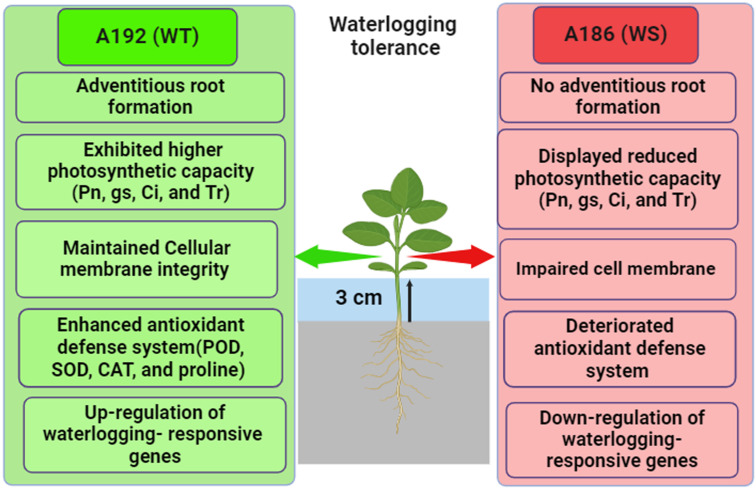
A schematic representation of possible mechanisms of waterlogging tolerance between A192 (WT) and A186 (WS) soybean lines. Green arrow indicates that the WT line exhibited higher tolerance to waterlogging stress, whereas the red arrow indicates that the WS line showed inferior tolerance. Pn: net photosynthesis rate; gs: stomatal conductance; Ci: net CO_2_ assimilation rate; Tr: transpiration rate; MDA: malondialdehyde; EL: electrolyte leakage; POD: peroxidase; SOD: superoxide dismutase; CAT: catalase. This figure was generated with the help of Biorender (https://biorender.com/).

Based on the current investigation’s findings, we propose a model for the mechanisms associated with waterlogging tolerance in soybean (Figure 9). Our findings demonstrated that both the WT and WS lines exhibited different response patterns at the morpho-physiological, biochemical, and transcriptional levels under waterlogging conditions; the WT line remained more stable during waterlogging. Waterlogging had less of an impact on plant growth in the WT line as the plants’ architecture remained intact and their green leaves were fully extended. Moreover, the WT line had a better photosynthetic performance, which resulted in a reduction in biomass and chlorophyll content under waterlogging. Furthermore, the WT line exhibited elevated activities of antioxidant enzymes and a higher accumulation of proline compared to the WS line. These attributes contribute to the protection of cell membranes and photosynthetic machinery from oxidative damage during the waterlogging treatment. Meanwhile, the WT line showed increased expression of waterlogging-responsive genes, suggesting that it possesses a stronger genetic foundation against waterlogging compared to the WS line. This study offers potential for the future improvement of varieties with enhanced tolerance to waterlogging.

## 5. Materials and Methods

### 5.1. Plant Materials and Waterlogging Conditions

Based on a preliminary screening of soybean lines for their response to waterlogging stress, two soybean introgression lines (ILs), A192 (waterlogging tolerant/WT) and A186 (waterlogging sensitive/WS), were selected from 70 tested ILs for their growth performance at the seedling stage under waterlogging stress. These lines were developed through three rounds of backcrosses from an NN86-4 (recurrent parent) × PI342618B cross. PI342618B is a wild soybean accession with high resistance to flooding stress [51]. Soybean seeds were acquired from the Soybean Improvement Center located at Nanjing Agricultural University in Nanjing, China. These lines were planted in 50-hole plastic trays (40 cm × 20 cm) filled with nutrient soil (Jiangsu Xingnong Substrate Technology Co., Ltd. Zhenjiang, China): vermiculite (1:1). The plants were grown at 28 °C (day)/24 °C (night) with a 14 h (light)/10 h (dark) photoperiod, under a light intensity of 20,000 lux. After germination, uniform and healthy seedlings were placed in plastic containers. The experiment was conducted at the vegetative (V1) stage, where control plants received regular amounts of water, while a 21-day waterlogging treatment was imposed by maintaining the water level at more than 3 cm above the soil surface. The experimental lines (WT and WS) were evaluated under both control and waterlogging conditions in a completely randomized design with three replications (5 plants per replication for each treatment). The soybean plant samples were frozen in liquid nitrogen and kept at −80 °C until they were needed for analysis.

### 5.2. Measurement of Morpho-Physiological Parameters

RL, ShL, RFW, ShFW, RDW, and ShDW were measured following the procedures outlined by Kim et al. [52]. Measurements for three plants per replicate were recorded.

The relative value of the total Chl content was determined using a Soil Plant Analysis Development (SPAD) chlorophyll meter (SPAD-502 plus, Konica Minolta Inc., Tokyo, Japan). The contents of different leaf pigments were also quantified according to a previously published protocol [53]. Leaf pigments were extracted from the upper leaves of both control and stressed plants [54]. A 0.1 g sample of fresh leaves was finely chopped and steeped in 80% acetone at room temperature for 24 h. The pigments of three different replicates were measured using a Tecan Infinite Pro Microplate Reader (Tecan Austria GmbH, Grodig, Austria). In order to prevent the deterioration of the photosynthetic pigments, all experiment operations were conducted in the absence of light.

The photosynthetic physiology parameters of Pn (μmol m^−2^ s^−1^), gs (mol m^−2^ s ^−1^), Ci (µmol mol^−1^), and Tr (mmol m^−2^ s^−1^) were assessed between 10:00 and 11:00 am from the third trifoliate leaf from the top using a LI-6400XT portable photosynthesis system (LI-COR Corporate, Lincoln, NE, USA). The Chl fluorescence parameters Fo (minimum fluorescence yield), Fm (maximum fluorescence yield), Fv (variable fluorescence), and Fv/Fm (maximum photochemical efficiency) were evaluated using an IMAGING-PAM Chl fluorescence analyzer (Heinz Walz, Effeltrich, Germany). Prior to measurement, the plants were allowed to adapt in darkness for 30 min.

Approximately 0.1 g of fresh leaf was utilized for measuring EL. Briefly, leaf segments were submerged in a 50 mL tube that contained 20 mL of deionized water, and the tube was then left at room temperature for 24 h in the dark. The initial electrical conductivity (EC1) was measured following incubation. The leaf segments in the 50 mL tube were heated for 20 min at 95 °C in a temperature-controlled water bath. After cooling, the electrical conductivity (EC2) was measured. The EL was computed as the EC1/EC2 percentage.

### 5.3. Estimation of Antioxidant Enzyme Activity

The activities of POD, SOD, and CAT, as well as MDA and proline content, were determined using a POD Assay Kit (A084-3), SOD Assay Kit (T-SOD, A001-1), CAT Assay Kit (A007-1), MDA Assay Kit (A003), and proline assay kit (A207), respectively, by following the manufacturer’s protocol (Nanjing Jiancheng Bioengineering Institute, Nanjing, China). Briefly, 1.0 g of a fresh leaf sample was grounded into slices using a mortar and pestle. These slices were then mixed with 9 mL of an ice-cold 20× phosphate-buffered saline solution (Beijing Solarbio Science & Technology Co., Ltd., Beijing, China) with a pH range of 7.2–7.3. The homogenates were further centrifuged at 3500 rpm for 10 min at 4 °C. The supernatants were collected and used as crude extracts for the assays, which were measured using a UV-1800 Shimadzu spectrophotometer (SHIMADZU Corporation, Kyoto, Japan).

### 5.4. H_2_O_2_ Detection

Following a couple of modifications from Kaur et al. [55], root samples of control and waterlogging stress plants were immersed in a 2′,7′-dichlorodihydrofluorescein diacetate (H_2_DCFDA) solution in order to detect hydrogen peroxide (H_2_O_2_). Briefly, root tips (approximately 1 cm) from both WT and WS lines were taken for the H_2_DCFDA assay. The root tips were then instantly dipped using tweezers into a 20 µM H2DCFDA (Sigma-Aldrich, St. Louis, MO, USA) solution on a Petri plate (35 mm). The dipped root samples were kept in a vacuum infiltration chamber for 20 min at a pressure of 60 KPa and then incubated at room temperature for 20 min in the dark. We washed the root samples three times with autoclaved double-distilled water to remove the excess H_2_DCFDA after incubation and dipped the samples in 20% glycerol after washing. The roots were then photographed using a Carl Zeiss HBO200 microscope (Carl Zeiss, Inc., New York, NY, USA).

### 5.5. RNA Extraction and Gene Expression Analysis Using Quantitative Real-Time PCR

Root samples from both the control and waterlogged treatments were used for total RNA extraction. The RNA extraction was performed using the RNA Prepare Plant Kit (TIANGEN, Beijing, China). To synthesize complementary DNA (cDNA), three plants from each biological replicate were used with the HiScript II Q RT superMix for qPCR (+gDNA wiper) kit as per the manufacturer’s instructions (Vazyme, Nanjing, China). Real-time quantitative gene amplification was carried out using the ChamQTM SYBR qPCR Master Mix (Vazyme, Nanjing, China) on a BIO-RAD system (CFX96TM Real-Time, Hercules, CA, USA). The PCR conditions consisted of an initial step of 95 °C for 3 min, followed by 40 cycles of 95 °C for 20 s, 60 °C for 30 s, and 65 °C for 5 s. The PCR reactions were normalized using the Ct value of GmActin11 (*Glyma.18g290800*), which was used as an internal control. Each control and treatment included three biological replicates and three technical replicates. The list of primers used for the qRT-PCR is shown in Table 2.

### 5.6. Statistical Analysis

The collected data on the plants’ morphology, biochemistry, and physiology were analyzed using IBM SPSS software version 23 (IBM Corp. Released 2015. IBM SPSS Statistics for Windows, Version 23.0. Armonk, NY, USA: IBM Corp). The analysis involved a one-way analysis of variance. Three replicates were used for each investigation, and the means were compared using Duncan’s Multiple Range Test. A value of *p* < 0.05 was considered statistically significant. The graphs were created using OriginPro 9.0 software (OriginLab Corporation, Northampton, MA, USA).

## Figures and Tables

**Table 1:** Comparison of morphological indicators in WT and WS soybean lines with and without waterlogging.

Time Point	Line	Treatment	Root Length (cm)	Shoot Length (cm)	Root Fresh Weight (g)	Shoot Fresh Weight (g)	Root Dry Weight (g)	Shoot Dry Weight (g)
21 DAT	WT	Control	30.40 ± 0.19 ^a^	75.20 ± 0.06 ^a^	6.51 ± 0.16 ^a^	13.43 ±.05 ^a^	1.16 ± 0.08 ^a^	3.68 ± 0.07 ^a^
		Waterlogged	27.18 ± 0.02 ^b^	70.17 ± 0.67 ^b^	4.88 ± 0.04 ^b^	11.23 ± 0.06 ^b^	0.88 ± 0.07 ^b^	2.55 ± 0.06 ^b^
	WS	Control	30.49 ± 0.08 ^a^	73.48 ± 0.38 ^a^	6.37 ± 0.36 ^a^	13.40 ± 0.03 ^a^	1.14 ± 0.05 ^a^	3.56 ± 0.05 ^a^
		Waterlogged	20.13 ± 0.10 ^c^	53.77 ± 0.82 ^c^	2.38 ± 0.14 ^c^	8.47 ± 0.03 ^c^	0.41 ± 0.02 ^c^	1.65 ± 0.02 ^c^

WT = waterlogging tolerant, WS = waterlogging sensitive, DAT = days after treatment. Different lower case letters indicate statistically significant differences according to Duncan’s Multiple Range Test (DMRT) with *p* < 0.05.

**Table 2:** Primer sequences for qRT-PCR analysis under control and waterlogging stress conditions.

Gene ID	Gene Name	Gene Function		Primer Sequence (5′->3′)
*Glyma.02G008900*	*POD1*	Peroxidase 5-like	F:	TGACACTGTCTGGAGCACAT
			R:	AAAGAGTACAAGCGGTCGGA
*Glyma.17G053000*	*POD2*	Peroxidase N-like	F:	TATTGGCCGAGCAAGGTGTA
			R:	ACTTTGCAGGTCAGAGAGCA
*Glyma.02G087700*	*FeSOD*	Superoxide dismutase (Fe)	F:	TGGTGAAGACTCCCAATGCT
			R:	TGACTGCATCCCAAGACACA
*Glyma.11G192700*	*Cu*/*ZnSOD*	Superoxide dismutase (Cu-Zn)	F:	CCCTTTCTCCGGTCATCCTT
			R:	CTTGGAGCGTGAAAGCGTTA
Glyma.04G017500	*CAT1*	Catalase	F:	TGGTCGCTTGGTCCTGAATA
			R:	GGTCCAAGTCTGTGCCTTTG
Glyma.17G261700	*CAT2*	Catalase	F:	ACAAGAATCGGCCATCAAGC
			R:	GCAAGCTTCTCCACCAGATG
*Glyma.04G245900*	*ACO1*	ACC oxidase 5	F:	GCGTCATCCTACTCCTCCAA
			R:	ACCATTGCTCAGGACCTCAA
*Glyma.09G255000*	*ACO2*	ACC oxidase homolog 1	F:	CCTAAGGCCTCCATTTGCAC
			R:	TCTCACTCTCCCACCTCTCA
*Glyma.05G223000*	*ACS1*	ACC synthase 7	F:	AACAGCAATGGCAAGCTTCA
			R:	AGAGCATCTCCTGGGTTAGC
*Glyma.08G030100*	*ACS2*	ACC synthase 7	F:	AACAGCAATGGCAAGCTTCA
			R:	GGCGTTGGAACAAGTAGAGC
*Glyma.18G290800*	*Actin11*	Reference gene	F:	CGGTGGTTCTATCTTGGCATC
			R:	GTCTTTCGCTTCAATAACCCTA

## Data Availability

The data that support the findings of this study are presented in the article.
